# Automated Urinal-Based Specific Gravity Measurement Device for Real-Time Hydration Monitoring in Male Athletes

**DOI:** 10.3389/fspor.2022.921418

**Published:** 2022-06-16

**Authors:** Brian F. Bender, Nick J. Johnson, Jasmine A. Berry, Kelvin M. Frazier, Michael B. Bender

**Affiliations:** Intake Health, Raleigh, NC, United States

**Keywords:** urinalysis, hydration, sports science, sports technology, wellness, safety, athletic performance

## Abstract

Acute and chronic hydration status is important for athlete safety and performance and is frequently measured by sports scientists and performance staff in team environments via urinalysis. However, the time required for urine collection, staff testing, and reporting often delays immediate reporting and personalized nutrition insight in situations of acute hydration management before training or competition. Furthermore, the burdensome urine collection and testing process often renders chronic hydration monitoring sporadic or non-existent in real-world settings. An automated urinalysis device (InFlow) was developed to measure specific gravity, an index of hydration status, in real-time during urination. The device was strongly correlated to optical refractometry with a mean absolute error of 0.0029 (±0.0021). Our results show this device provides a novel and useful approach for real-time hydration status via urinalysis for male athletes in team environments with high testing frequency demands.

## Introduction

Water is essential for life, playing such vital physiological roles as a cellular and tissue building material, a solvent and reaction medium, a carrier of nutrients and waste, and a medium for thermoregulation and shock absorption (Jéquier and Constant, 2010). As dehydration ensues and leads to a state of hypohydration, negative impacts on blood flow, skeletal muscle metabolism, cardiovascular strain, and thermoregulation often lead to impaired physiological function and athletic performance such as a shorter time to exhaustion and lower exercise intensity (Cheuvront and Kenefick, 2014). This is particularly true for athletes and other highly active individuals, where sweat output is high and performance optimization is a top priority (Sawka et al., 2007b). It is well-known that dehydration impairs aerobic performance and is increasingly demonstrating impairment in areas of strength and power, cognitive function, mood, and sleep (Cheuvront and Kenefick, 2014; Harris et al., 2019; Deshayes et al., 2020). Maintenance of euhydration has been stressed for endurance athletes. However, a state of hypohydration has been shown to negatively affect skill-based performance metrics in sports such as soccer (McGregor et al., 1999; Edwards et al., 2007) and basketball (Baker et al., 2007a,b).

The extreme importance of euhydration on preserving organ function and health has resulted in the evolution of sensitive and precise homeostatic mechanisms to maintain fluid and electrolyte balance and results in physiological changes that have been used as biomarkers of hydration status (Jéquier and Constant, 2010). One regulatory mechanism is related to thirst, generated via a neuroendocrine response to the osmotically driven shrinking of cells when water deficits result in intracellular water leaving the cell to dilute an overly ionic extracellular fluid space (Cheuvront and Kenefick, 2014; Leib et al., 2016). Another physiological mechanism triggered during intracellular volume contraction is signaling from the antidiuretic hormone vasopressin, triggering the kidneys to produce a smaller volume of more concentrated urine (Popkin et al., 2010). This unique role of the kidneys to regulate blood osmolality is what has led to the use of several urine indices as biomarkers of hydration status, including urine osmolality, urine specific gravity (USG), 24-h urine volume, urine color, and urine conductivity (Armstrong et al., 1994).

A more concentrated urine sample, as indicated by a higher urine osmolality, higher urine specific gravity, lower 24-h urine volume, darker urine color, or higher urine conductivity, correlates to other commonly used biomarkers of hypohydration status such as blood plasma osmolality and body mass decrease. Urine osmolality is typically measured via freezing point depression and represents the concentration of all solutes in solution. Urine specific gravity measures the density of the urine solution relative to water and thus heavier solutes, such as glucose and creatinine, can bias the results. Urine color is often measured via comparison to color charts and can be a quick and easy method but is subject to user error and some potential confounding physiological conditions or presence of supplements. Urine conductivity is a function of conductive species in solution, largely sodium, and correlates to total solute concentration. All these techniques trend together. However, no individual measurement can provide a complete picture of hydration status, nor can each be reliable in all individuals and for all use cases. For example, a rugby player with exceptionally high lean body mass typically excretes higher rates of larger molecules like creatinine that bias urine specific gravity toward the higher end of the scale, suggesting a more hypohydrated state when compared to leaner runners, despite similar blood plasma osmolality measurements (Hamouti et al., 2010). Furthermore, low Index of Individuality (II) for several of these biomarkers (Cheuvront et al., 2010) leaves the need for repeated testing and individual baselining important for better assessing dynamic hydration status (Cheuvront et al., 2011).

Measuring athlete hydration status in real-world settings is often difficult, necessitating a balance between accuracy, cost, and ease-of-use (Belval et al., 2019). Methods for measuring hydration have been reviewed elsewhere, including their benefits and limitations (Barley et al., 2020). For example, body mass change and bioelectrical impedance analysis (BIA) are non-invasive and relatively simple (players only need to stand on the device for a few moments). However, as with all hydration assessment techniques (Armstrong, 2007), these methods possess limitations. Confounding activities include recent food ingestion, fluid ingestion, urination, defecation, and intensive physical activity (Mialich et al., 2014). In addition, due to both logistical challenges and reliability, USG is generally recommended over BIA in athletic settings for serial hydration assessment (Barley et al., 2020). The current processes most often used for urine-testing are manual, requiring players to urinate into cups which are later collected by staff that perform dipstick or optical refractometer testing. This is both labor and time-intensive and results in infrequent testing and/or delayed reporting. Optimal solutions are often dubbed “invisible monitoring,” which require no athlete burden and facilitate buy-in (Windt et al., 2020). In addition, some of the best results for player optimization of health and safety comes from player empowerment that drives self-regulation (Kim and Cruz, 2021). An automated, accurate USG measurement device that allows players to self-monitor hydration status could provide high-compliance testing and improved hydration awareness.

## Materials and Methods

### Study Design

The study was carried out in February 2022 at three public U.S. University athletic training facilities. All measurements were performed using surplus human urine samples to requirement (≥50 mL) from routine testing. Anonymized samples were used for all experiments. Samples were stored at room temperature, did not undergo any processing or centrifugation, and were analyzed within 2 h of sample collection.

The use of patient samples complied with all relevant national regulations and institutional policies. The study does not conform to NIH definition of a Clinical Trail per NOT-OD-15-015. In addition, the study does not conform to the definition of human subjects research per 45 CFR 46, as only unidentifiable surplus samples from routine testing were used in the study.

Participants included 151 NCAA male football athletes from three collegiate institutions. Urine samples were collected into plastic cups by each player and brought to performance staff and dietitians during the normal course of their activities for USG testing using manual (Teckoplus) and digital optical refractometers (MISCO Palm Abbe and Atago 3741 PEN) and dipsticks (Diagnox Urinox-10) for a subset of the tests. After normal testing, the surplus urine samples were poured through the InFlow system.

### InFlow System

**Figure 1:**
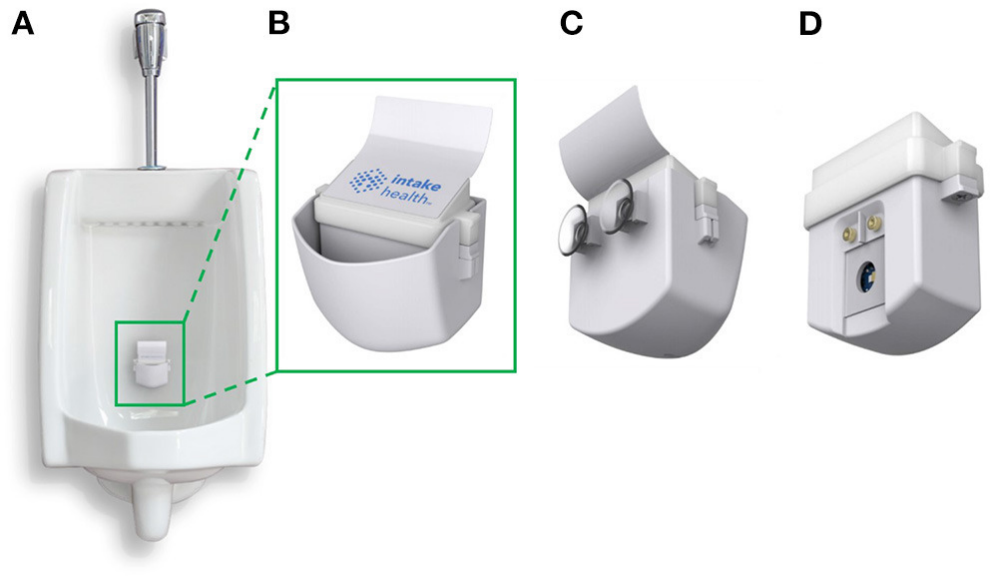
The InFlow Urinalysis System. **(A)** Fully assembled system installed in a urinal using rear suction cups capable of catching a stream of urine in real-time. Urine fills the cup at a rate faster than it can drain through a hole in the bottom. **(B)** The front, fully assembled InFlow system magnified. LED lights under the top of the insert housing flash red, yellow, or green to categorize results for the user as dehydrated, mildly dehydrated, and hydrated, respectively. **(C)** Rear view of the full assembly showing suction cups for installation. **(D)** Rear view of the removed insert sub-assembly showing the sensors that interface with the urine sample during use. As urine fills the cup, fluid engulfs the insert from the bottom. The metal electrodes register the presence of fluid by shorting the pin voltage and initiate testing via the LED and photodiodes residing behind a glass window.

The InFlow system is designed to capture urine in real-time during a urination event from a urinal (Figure 1A). The system has a cup to easily catch and fill with urine (Figure 1B). The system is installed by pressing the unit against the wall of a urinal using the suction cups on the back of the device (Figure 1C). During urination, urine quickly fills the cup volume faster than it can drain through a small hole in the bottom of the cup. A removable insert housed within the cup (Figure 1D) holds the electronics, sensors, and power. As the cup fills, the fluid covers the testing chamber, turning on the system and performing a test in <2 s.

### Analytical Imprecision and Bias

For analytical testing and quantitative analysis of the InFlow system, the mean (μ) and standard deviation (SD) were calculated. Method comparison results for the InFlow system were assessed using Bland-Altman difference plots and regression analysis (including Pearson's r correlation coefficients) for quantitative parameters (USG). Confidence intervals and prediction intervals at 95% were calculated for InFlow performance against the manual optical refractometer.

Analytical system performance was assessed using artificial urine control (Aldon Life Sciences, IS5070). The SD and coefficient of variation (CV%) (SD/μ × 100) of total imprecision were calculated by testing artificial urine control across 10 units in triplicate. The within-unit SD was calculated as the average SD across triplicate back-to-back runs from the same unit across 10 units. The between-unit SD was calculated using artificial urine control across 10 units. For each pool, the “observed” reference USG value was established for each specimen using a manual optical refractometer and taking the mean USG. The InFlow system mean and SD are derived from measurements through the urinalysis device. Data was analyzed using the Westgard model, using Total Error (TE) and TE (%) defined by Equations (1) and (2), respectively. The threshold used for acceptable percent Total Allowable Error (TAE%) for USG was ±0.6% (Ricós et al., 1999). Results provided from analytical sensitivity experiments were rounded to 4 decimal places, except TE (%) which was rounded to 2 decimal places.


(1) $$\begin{array}{rcl} Total\;Error\;\left(TE\right) & = & |Bias|+2SD \end{array}$$

(2) $$\begin{array}{rcl} TE\left(\%\right) & = & \left(TE÷reference\;mean\right)\times 100 \end{array}$$

### Diagnostic Performance

Assessment of classification of dehydration was performed at a criterion value (USG ≥ 1.020) designated by the American College of Sports Medicine (ACSM) and the National Athletic Training Association (NATA) (Casa et al., 2000; Sawka et al., 2007a). A positive result was assigned to a dehydrated sample and a negative result was assigned to a euhydrated sample. True Positives (TP) were assigned to samples the InFlow system classified as a positive result when the manual optical refractometer reported a positive result, and True Negatives (TN) were assigned to samples the InFlow system classified as a negative result when the manual optical refractometer reported a negative result. In contrast, False Positives (FP) were assigned to samples the InFlow system classified as a positive result when the manual optical refractometer reported a negative result, and False Negatives (FN) were assigned to samples the InFlow system classified as a negative result when the manual optical refractometer reported a positive result. Diagnostic accuracy, sensitivity, specificity, and precision were calculated (Zweig and Campbell, 1993).

Receiver operator characteristic (ROC) analysis was performed to assess diagnostic accuracy represented by the area under the ROC curve (AUC) (Zweig and Campbell, 1993). There is no established analytical goal for dehydration; a recommended minimum of 80% for sensitivity and specificity was used (Zweig and Campbell, 1993; Cheuvront et al., 2010), which would represent odds of 4 to 1 in favor of a correct classification.

## Results

The distribution of USG values among the sample population ranged from 1.003 to 1.036 (Supplementary Figure 1). The mean USG was 1.018 (±0.009) and approximately 45% of samples tested were hypohydrated (USG ≥1.020). This distribution USG mean is similar to population USG means of other athletes (1.018 ± 0.009) prior to exercise (Stover et al., 2006). The percentage of hypohydrated samples is less but similar to (66%) other NCAA athlete samples (Volpe et al., 2009) and the range and distribution (SD) provide an adequate and representative array of urine samples for system performance evaluation.

The InFlow system's design was chosen to minimize testing burden on the user while maintaining adequate accuracy for hydration reporting consistent with existing protocols and testing equipment. Results from precision studies are shown in Table 1. Very low total imprecision was demonstrated using artificial urine control material. Imprecision estimates (within unit, between unit) were estimated in USG “units.” The within-unit imprecision of 0.0001 is comparable to digital optical refractometer resolution (0.0001) (Atago, 2022; MISCO, 2022).

**Table 1:** Imprecision of InFlow system measured in USG units using artificial urine control.

**Material**	**Mean USG**	**Total imprecision (SD)**	**Total imprecision (%CV)**	**Within unit (SD)**	**Between unit (SD)**
Artificial urine control	1.0325	0.0009	0.09%	0.0001	0.0009

**Figure 2:**
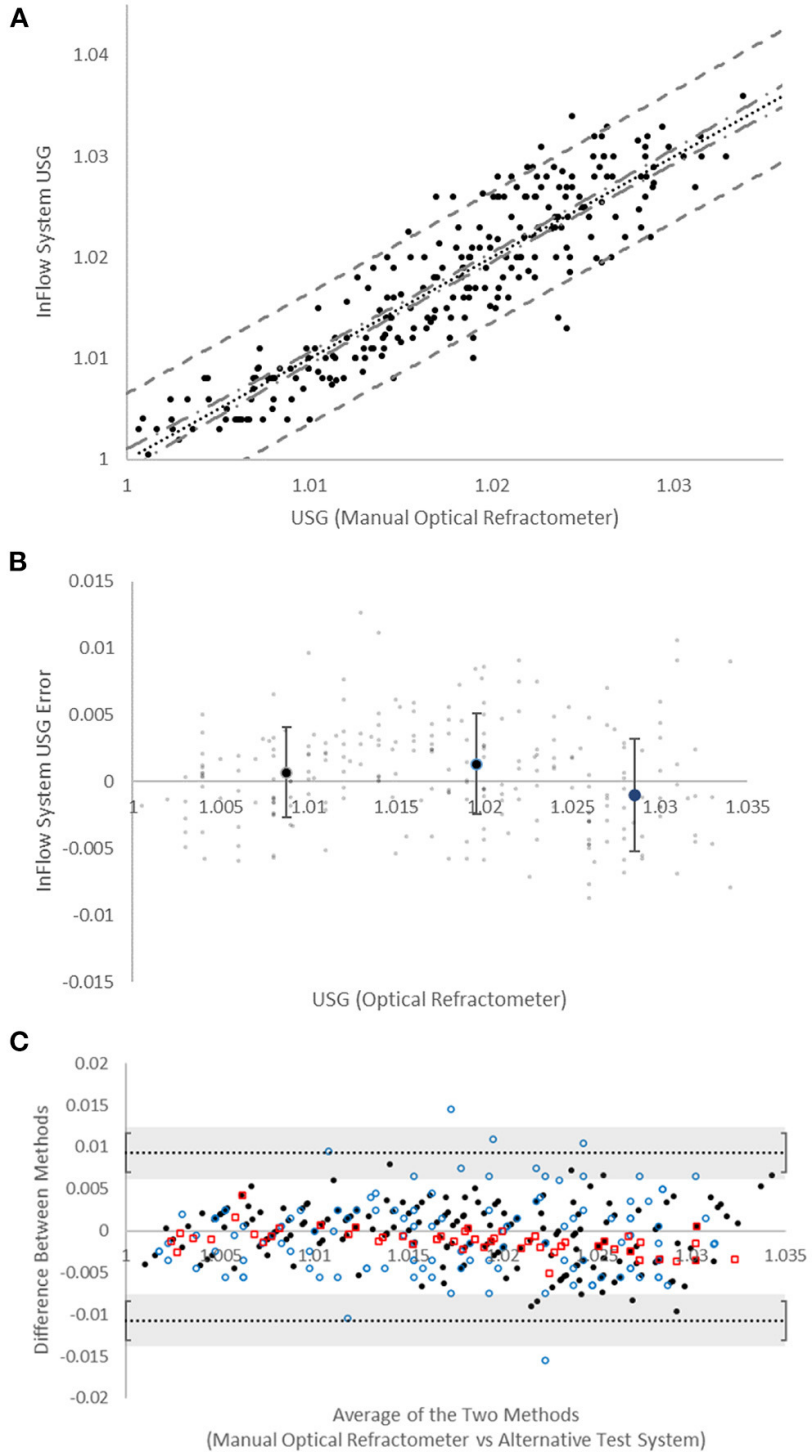
Device accuracy. **(A)** Comparison of manual optical refractometer (x-axis) versus InFlow system (y-axis) results. Dotted line (^…^) is linear regression (*r* = 0.90). Dot-dash line (**-** ^**.**^ **-**) represents 95% confidence interval. Dashed line (- -) represents 95% prediction interval. **(B)** Bias (in USG “units”) of InFlow system vs. manual optical refractometry. Large dots represent averages at each USG range (<1.015, 1.015−1.025, >1.025). Error bars represent SD. Small dots represent individual test results. **(C)** Bland-Altman plots of agreement between the manual optical refractometer with the InFlow system (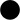), the MISCO digital optical refractometer (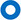), and the Atago digital optical refractometer (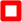). All systems fall with the agreement limits set at the reference change value (0.010) for USG established via *CV*_*I*_ and *CV*_*G*_ (Cheuvront et al., 2010, 2011). Error bars represent the SD of the agreement limits.

Analytical performance studies (Figure 2A) demonstrated strong correlation to manual optical refractometry (*r* = 0.90; *n* = 247 specimens, USG range 1.003–1.036). The mean population error (Figure 2B) was not significantly different than zero at any USG range with root mean squared error (RMSE) of 0.0036. The mean absolute error (±SD) as a function of USG (0.0029 ± 0.0022) tended to trend in a positive direction but this trend was not significant (Supplementary Figure 2). The error between the InFlow system and a manual optical refractometer was compared against the error between digital optical refractometers and the manual optical refractometer via Bland-Altman plot of agreement (Figure 2C). All points representing error between InFlow results and the manual optical refractometer fell between the limits of agreement established by clinically relevant USG thresholds based on inter- and intraindividual variability for hydration assessment (Cheuvront et al., 2010, 2011).

Analytical imprecision and bias testing demonstrated acceptable TE (%) [defined as <0.6% TAE(%)] at all USG levels tested (Table 2). The analytical imprecision, *CV*_*A*_, of 0.09% was below half the intraindividual variation, *CV*_*I*_, for USG (0.4%), as recommended in clinical chemistry best practices (Fraser and Harris, 1989; White et al., 2004; Cheuvront et al., 2010).

**Table 2:** Analytical sensitivity of Inflow system by USG range as measured against manual optical refractometry.

**USG range**	**Number of samples in set**	**Observed mean of sample set**	**InFlow mean of samples**	**InFlow SD**	**Bias**	**TE (%)**
<1.015	96	1.0088	1.0095	0.0023	0.0007	0.53
1.015–1.025	85	1.0196	1.0209	0.0023	0.0013	0.59
>1.025	66	1.0286	1.0276	0.0025	−0.0010	0.59

Diagnostic performance evaluation yielded an accuracy of 87%, a sensitivity of 87%, a specificity of 88%, and a precision of 85% (TP = 96; TN = 120; FP = 17; FN = 14). These values exceed cutoff values for sensitivity and sensitivity of 70% used elsewhere for urine-based hydration classification at 1.020 (Hooper et al., 2016) and our own analytical goal of 80% (Cheuvront et al., 2010). These assessments demonstrate the InFlow system performs adequately for USG testing for hydration assessment given USG's inter- and intraindividual variability. ROC analysis produced an AUC of 0.94, providing evidence of generally acceptable diagnostic accuracy (Supplementary Figure 3).

## Discussion

Experiments demonstrated acceptable analytical and diagnostic performance of the automated InFlow system for USG, including imprecision and accuracy. The instrument worked reliably with sample flowing through the system, which is analogous to real-world use in a urinal. We did not encounter ambient lighting-related difficulties (which others have reported with digital optical refractometers) (Minton et al., 2015), because the InFlow system measurement chamber is internally housed and shielded from external lighting conditions. No staining of the glass window occurred during the study or during prolonged benchtop testing of over 45 days of testing. Power analyses demonstrated a low sleep current while not in use of around 10 μA and a test current output of approximately 0.41 mAh (Supplementary Figure 4). The InFlow system performed over 5,000 tests per charge, similar to the digital optical refractometers tests. The InFlow system includes a wireless Qi charging system for simple battery recharging.

The InFlow system was compared to manual optical refractometry for error analysis. The mean absolute error (±SD) between manual optical refractometry and the InFlow system was 0.0029 (±0.0021). To compare these results to other available tools for measuring USG, two digital optical refractometers were compared to manual optical refractometry (Supplementary Figure 5). The mean absolute error of the MISCO digital optical refractometer (0.0038 ± 0.0047) was higher than the InFlow system, but the mean absolute error of the Atago digital optical refractometer (0.0016 ± 0.0011) was lower than the InFlow system. All three systems were deemed interchangeable for use in hydration assessment via USG in a sports environment based on Bland-Altman analysis (Figure 2C). Based on recommendations for setting Bland-Altman agreement limits on biologically and analytically relevant criteria (Giavarina, 2015), limits of ±0.010 were used as defined by the reference change value for USG-based hydration assessment given its intraindividual (*CV*_*I*_ = 0.4) and interindividual (*CV*_*G*_ = 1.0) variability (Cheuvront et al., 2010, 2011). These results generally point to interchangeability between the digital optical refractometers and the InFlow system for use in USG reporting (Giavarina, 2015). All datapoints fell within the limits with the exception of 4% (*n* = 5) of MISCO digital optical refractometer readings.

Urine dipstick testing has known error associated with manual color comparison, lighting variation, sample size variation, timing variation, and the inherent variability associated with USG binning by 0.005 USG unit increments (de Buys Roessingh et al., 2001; Smith et al., 2017). A subset of urine samples (*n* = 119) was randomly selected for urine dipstick analysis. USG error (±SD) for dipstick testing compared against manual optical refractometry was 0.0051 (±0.0047) with *r* = 0.76 (Supplementary Figure 6). This error was significantly higher than the InFlow system (*p* < 0.001; α = 0.05).

Although there are a number of commercially available handheld digital optical refractometers including Palm Abbe (MISCO); PEN, UG-α, PAL-10S (Atago); Clinic-Chek, USG-Check, and TS METER D (Reichert; Depew, NY), this system represents the first automated device designed to measure USG from a urinal in real-time as the individual urinates into the system. This represents a significant improvement to USG testing in high frequency testing environments such as those found within collegiate and professional athletic programs. Testing time is a significant hurdle to large-scale, frequent hydration testing in team settings. This leads to delayed action. It is not uncommon for players to already leave the locker room and begin training or competition before hydration results have been measured, assessed, and reported. Similarly, the high burden on performance staff, necessitating tracking down players, handling urine cups, labeling, testing, and reporting, leads to a sporadic testing schedule. The InFlow system significantly reduces, and at times eliminates, the testing and reporting time by analyzing results in real-time during the act of urination and reporting those results directly to the player instantaneously.

The InFlow system provides significant improvement over manual quality control (QC) errors common to clinical testing procedures. Pre-analytical errors typically account for most QC errors and include mislabeling of sample containers and sorting errors (Delanghe and Speeckaert, 2014). Similarly, post-analytical errors such as mistakes in data transcription are common (Hammerling, 2012). InFlow's automated testing framework eliminates the need for sample collection, labeling, and data transcription and thus reduces, or eliminates, these common QC errors.

Future areas of research may include assessment of varying physiological and environmental conditions that present in altered urine color, such as conditions like rhabdomyolysis or medication/supplement use. Future research will also compare the InFlow system to other markers of hydration, such as blood and urine osmolality, alongside mechanistic studies in urine composition to improve accuracy and error reporting such as albumin (known to present during intense physical exercise) and creatinine (known to exist in higher concentrations in individuals with high lean muscle mass). In addition, broadening and diversifying the sample of users may improve the test statistics. Similarly, this system may be assessed for useability and accuracy in other environments that may provide benefit such as within industrial settings, military settings, and general consumer-facing health and wellness settings. Finally, altered designs for use among female athletes is another area of ongoing research.

In conclusion, the automated InFlow system was demonstrated to be a fast, simple, and accurate way to measure USG. The InFlow system met accuracy requirements for reliably monitoring USG for hydration assessment given its biological variation within an automated testing platform for male users from a urinal measured during the act of urination. The results from this report may prove valuable for those interested in evaluating use of the InFlow system in a variety of settings and applications in long-term, longitudinal hydration monitoring and behavior change studies.

## Data Availability Statement

The raw data supporting the conclusions of this article will be made available by the authors, without undue reservation.

## Author Contributions

BB conceived the analytical approach, derived the algorithms, analyzed the data, and wrote and edited the manuscript. BB, NJ, and MB conceived the overall system design, built and tested prototype hardware and firmware, and performed sample testing. MB assisted in field test data collection. JB assisted in statistical data analysis and manuscript editing. KF assisted with manuscript editing. All authors contributed to the article and approved the submitted version.

## Funding

This work was supported in part by funding from the National Science Foundation under grant 2026127. The views and opinions of the authors do not reflect those of the National Science Foundation.

## Conflict of Interest

The authors declare that they are employees of Bender Tech, LLC (dba Intake Health) and receive salaries through their positions with the company.

## Publisher's Note

All claims expressed in this article are solely those of the authors and do not necessarily represent those of their affiliated organizations, or those of the publisher, the editors and the reviewers. Any product that may be evaluated in this article, or claim that may be made by its manufacturer, is not guaranteed or endorsed by the publisher.

## References

[B1] Armstrong L. E. (2007). Assessing hydration status: the elusive gold standard. J. Am. Coll. Nutr. 26, 575S−584S. 10.1080/07315724.2007.10719661 17921468

[B2] Armstrong L. E. Maresh C. M. Castellani J. W. Bergeron M. F. Kenefick R. W. LaGasse K. E.. (1994). Urinary indices of hydration status. Int. J. Sport Nutr. 4, 265–279. 10.1123/ijsn.4.3.265 7987361

[B3] Atago (2022). Clinical Refractometers. Available online at: https://www.atago.net/USA/products_clinical.php

[B4] Baker L. B. Conroy D. E. Kenney W. L. (2007a). Dehydration impairs vigilance-related attention in male basketball players. Med. Sci. Sports Exerc. 39, 976–983. 10.1097/mss.0b013e3180471ff2 17545888

[B5] Baker L. B. Dougherty K. A. Chow M. Kenney W. L. (2007b). Progressive dehydration causes a progressive decline in basketball skill performance. Med. Sci. Sports Exerc. 39, 1114–1123. 10.1249/mss.0b013e3180574b02 17596779

[B6] Barley O. R. Chapman D. W. Abbiss C. R. (2020). Reviewing the current methods of assessing hydration in athletes. J. Int. Soc. Sports Nutr. 17, 52. 10.1186/s12970-020-00381-6 33126891 PMC7602338

[B7] Belval L. N. Hosokawa Y. Casa D. J. Adams W. M. Armstrong L. E. Baker L. B.. (2019). Practical hydration solutions for sports. Nutrients 11, 1550. 10.3390/nu11071550 31324008 PMC6682880

[B8] Casa D. J. Armstrong L. E. Hillman S. K. Montain S. J. Reiff R. Rich B. S.. (2000). National athletic trainers' association position statement: fluid replacement for athletes. J. Athletic Train. 35, 212–224. 16558633 PMC1323420

[B9] Cheuvront S. N. Ely B. R. Kenefick R. W. Sawka M. N. (2010). Biological variation and diagnostic accuracy of dehydration assessment markers. Am. J. Clin. Nutr. 92, 565–573. 10.3945/ajcn.2010.29490 20631205

[B10] Cheuvront S. N. Fraser C. G. Kenefick R. W. Ely B. R. Sawka M. N. (2011). Reference change values for monitoring dehydration. Clin. Chem. La. Med. 49, 1033–1037. 10.1515/CCLM.2011.170 21428854

[B11] Cheuvront S. N. Kenefick R. W. (2014). Dehydration: physiology, assessment, and performance effects, in Comprehensive Physiology, ed Terjung R. (Hoboken, NJ: John Wiley & Sons, Inc.), 257–285. 10.1002/cphy.c130017 24692140

[B12] de Buys Roessingh A. S. Drukker A. Guignard J. P. (2001). Dipstick measurements of urine specific gravity are unreliable. Arch. Dis. Childhood 85, 155–157. 10.1136/adc.85.2.155 11466191 PMC1718890

[B13] Delanghe J. Speeckaert M. (2014). Preanalytical requirements of urinalysis. Biochem. Med. 24, 89–104. 10.11613/BM.2014.011 24627718 PMC3936984

[B14] Deshayes T. A. Jeker D. Goulet E. D. B. (2020). Impact of pre-exercise hypohydration on aerobic exercise performance, peak oxygen consumption and oxygen consumption at lactate threshold: a systematic review with meta-analysis. Sports Med. 50, 581–596. 10.1007/s40279-019-01223-5 31728846

[B15] Edwards A. M. Mann M. E. Marfell-Jones M. J. Rankin D. M. Noakes T. D. Shillington D. P. (2007). Influence of moderate dehydration on soccer performance: physiological responses to 45 min of outdoor match-play and the immediate subsequent performance of sport-specific and mental concentration tests. Brit. J. Sports Med. 41, 385–391. 10.1136/bjsm.2006.033860 17272311 PMC2465308

[B16] Fraser C. G. Harris E. K. (1989). Generation and application of data on biological variation in clinical chemistry. Crit. Rev. Clin. Lab. Sci. 27, 409–437. 10.3109/10408368909106595 2679660

[B17] Giavarina D. (2015). Understanding Bland Altman analysis. Biochem. Med. 25, 141–151. 10.11613/BM.2015.015 26110027 PMC4470095

[B18] Hammerling J. A. (2012). A review of medical errors in laboratory diagnostics and where we are today. Lab. Med. 43, 41–44. 10.1309/LM6ER9WJR1IHQAUY

[B19] Hamouti N. del Coso J. Ávila A. Mora-Rodriguez R. (2010). Effects of athletes' muscle mass on urinary markers of hydration status. Eur. J. Appl. Physiol. 109, 213–219. 10.1007/s00421-009-1333-x 20058021

[B20] Harris P. R. Keen D. A. Constantopoulos E. Weninger S. N. Hines E. Koppinger M. P.. (2019). Fluid type influences acute hydration and muscle performance recovery in human subjects. J. Int. Soc. Sports Nutr. 16, 15. 10.1186/s12970-019-0282-y 30947727 PMC6449982

[B21] Hooper L. Bunn D. K. Abdelhamid A. Gillings R. Jennings A. Maas K.. (2016). Water-loss (intracellular) dehydration assessed using urinary tests: how well do they work? Diagnostic accuracy in older people. Am. J. Clin. Nutr. 104, 121–131. 10.3945/ajcn.115.119925 27225436

[B22] Jéquier E. Constant F. (2010). Water as an essential nutrient: the physiological basis of hydration. Eur. J. Clin. Nutr. 64, 115–123. 10.1038/ejcn.2009.111 19724292

[B23] Kim H.-D. Cruz A. B. (2021). Psychological influence of self-management on exercise self-confidence, satisfaction, and commitment of martial arts practitioners in Korea: a meta-analytic approach. Front. Psychol. 12, 691974. 10.3389/fpsyg.2021.691974 34135839 PMC8200399

[B24] Leib D. E. Zimmerman C. A. Knight Z. A. (2016). Thirst. Curr. Biol. 26, R1260–R1265. 10.1016/j.cub.2016.11.019 27997832 PMC5957508

[B25] McGregor S. J. Nicholas C. W. Lakomy H. K. (1999). The influence of intermittent high-intensity shuttle running and fluid ingestion on the performance of a soccer skill. J. Sports Sci. 17, 895–903. 10.1080/026404199365452 10585169

[B26] Mialich M. S. Maria J. Sicchieri F. Afonso A. Junior J. (2014). Analysis of body composition : a critical review of the use of bioelectrical impedance analysis. Int. J. Clin. Nutr. 2, 1–10. 10.12691/ijcn-2-1-125117995

[B27] Minton D. M. O'Neal E. K. Torres-McGehee T. M. (2015). Agreement of urine specific gravity measurements between manual and digital refractometers. J. Athletic Train. 50, 59–64. 10.4085/1062-6050-49.3.47 25280126 PMC4299737

[B28] MISCO (2022). Human Urine Refractometer Specifications. Available online at: https://www.misco.com/product/human-urine-refractometer-specific-gravity-total-solids-pa202x-093-095/

[B29] Popkin B. M. D'Anci K. E. Rosenberg I. H. (2010). Water, hydration, and health. Nutr. Rev. 68, 439–458. 10.1111/j.1753-4887.2010.00304.x 20646222 PMC2908954

[B30] Ricós C. Alvarez V. Cava F. García-Lario J. v. Hernández A.. (1999). Current databases on biological variation: pros, cons and progress. Scand. J. Clin. Lab. Investig. 59, 491–500. 10.1080/00365519950185229 10667686

[B31] Sawka M. N. Burke L. M. Eichner E. R. Maughan R. J. Montain S. J. Stachenfeld N. S. (2007a). Exercise and fluid replacement. Med. Sci. Sports Exerc. 39, 377–390. 10.1249/mss.0b013e31802ca597 17277604

[B32] Sawka M. N. Burke L. M. Eichner E. R. Maughan R. J. Montain S. J. Stachenfeld N. S. (2007b). American College of Sports Medicine position stand. Exercise and fluid replacement. Med. Sci. Sports Exerc. 39, 377–390. 17277604 10.1249/mss.0b013e31802ca597

[B33] Smith G. T. Dwork N. Khan S. A. Millet M. Magar K. Javanmard M.. (2017). Reducing user error in dipstick urinalysis with a low-cost slipping manifold and mobile phone platform (Conference Presentation), in Proc. SPIE. (San Francisco, CA). 10.1117/12.2256593

[B34] Stover E. A. Petrie H. J. Passe D. Horswill C. A. Murray B. Wildman R. (2006). Urine specific gravity in exercisers prior to physical training. Appl. Physiol. Nutr. Metab. 31, 320–327. 10.1139/h06-004 16770361

[B35] Volpe S. L. Poule K. A. Bland E. G. (2009). Estimation of prepractice hydration status of national collegiate athletic association division I athletes. J. Athlet. Train. 44, 624–629. 10.4085/1062-6050-44.6.624 19911089 PMC2775364

[B36] White G. H. Farrance I. AACB Uncertainty of Measurement Working Group (2004). Uncertainty of measurement in quantitative medical testing: a laboratory implementation guide. Clin. Biochem. Rev. 25, S1–S24. 18650962 PMC1934961

[B37] Windt J. MacDonald K. Taylor D. Zumbo B. D. Sporer B. C. Martin D. T. (2020). “To tech or not to tech?” A critical decision-making framework for implementing technology in sport. J. Athlet. Train. 55, 902–910. 10.4085/1062-6050-0540.19 32991702 PMC7534935

[B38] Zweig M. H. Campbell G. (1993). Receiver-operating characteristic (ROC) plots: a fundamental evaluation tool in clinical medicine. Clin. Chem. 39, 561–577. 10.1093/clinchem/39.4.561 8472349

