# Supplementary material for: Automated Urinal-Based Specific Gravity Measurement Device for Real-Time Hydration Monitoring in Male Athletes
Source: Front Sports Act Living. 2022 Jun 16;4:921418. doi: 10.3389/fspor.2022.921418 (PMC9243503; doi:10.3389/fspor.2022.921418)
Supplement: Supplementary file 1 [file Data_Sheet_1.pdf]

Supplementary Materials

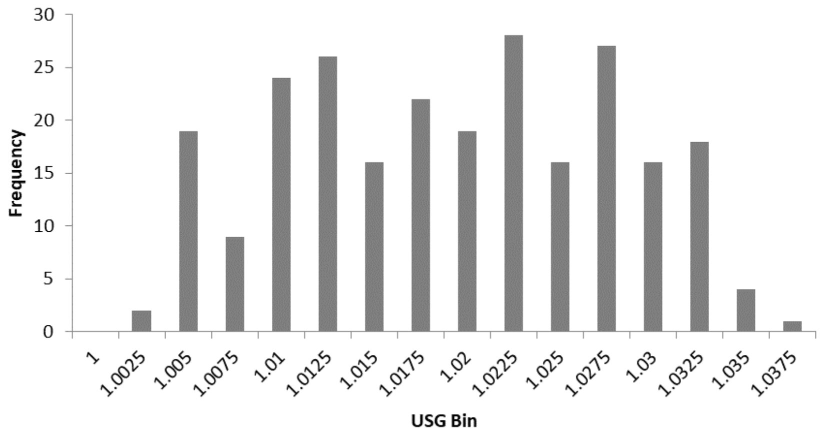

Supplementary Figure 1: Population USG histogram. Distribution of USG values across the test population. Range: 1.003 ≤ USG ≤ 1.036; Mean (SD): USG = 1.018 (± 0.009).

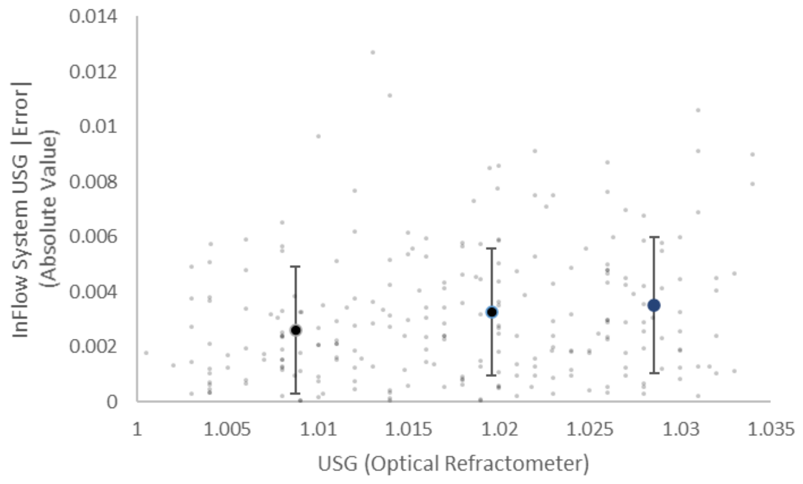

Supplementary Figure 2: Absolute value of error of InFlow system. The magnitude of the error by USG had a non-significant upward trend by USG.

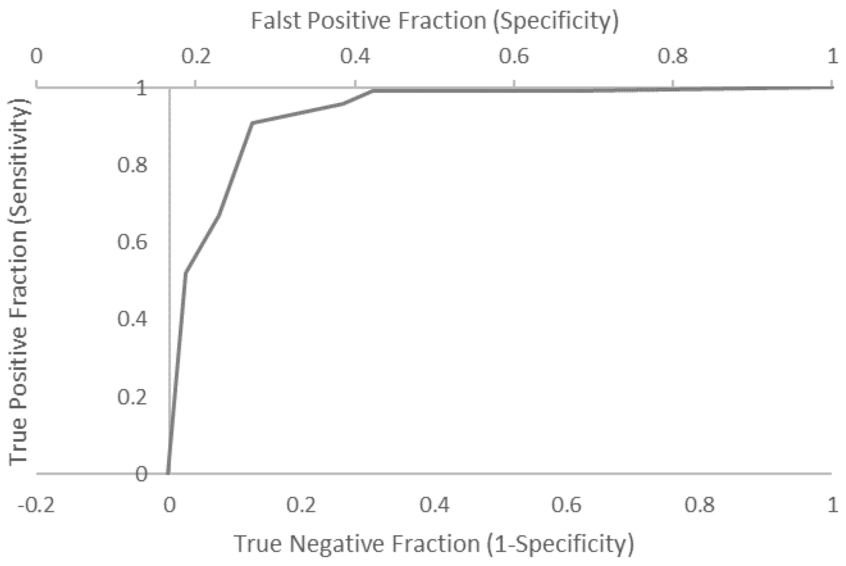

Supplementary Figure 3: ROC analysis demonstrating diagnostic performance of InFlow system with AUC=0.94.

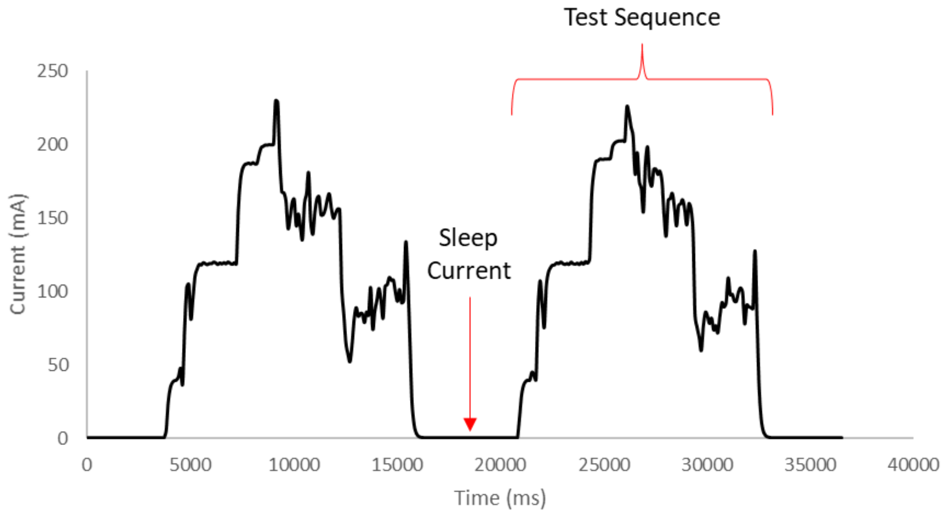

Supplementary Figure 4: Power analysis. Current measured every 100 ms during sleep mode between tests and during two separate tests. Sleep current averages approximately 11 µA, while a testing sequence uses approximately 0.41 mAh.

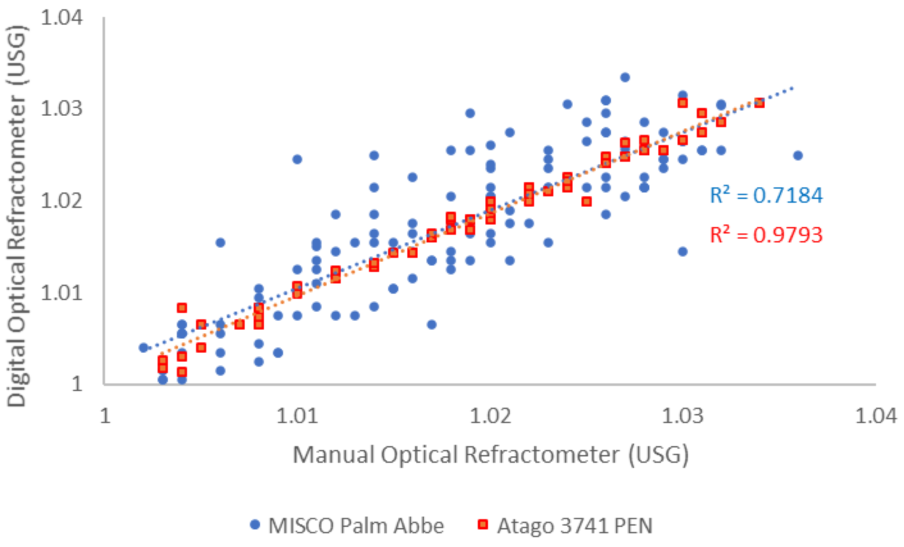

Supplementary Figure 5: Correlations between manual optical refractometer and digital optical refractometers tested. Blue circles (●) represent measurements using the MISCO Palm Abbe. Red squares (■) represent measurements using the Atago 3741 PEN.

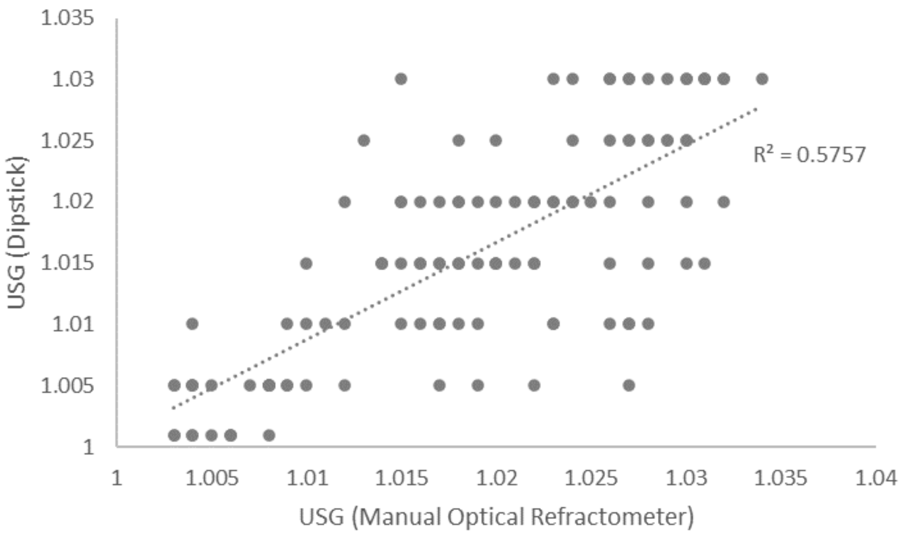

Supplementary Figure 6: Dipstick analysis. Correlation between manual optical refractometer and dipstick testing of USG on subset of samples (n=119).
